# What is the right sequencing approach? Solo VS extended family analysis in consanguineous populations

**DOI:** 10.1186/s12920-020-00743-8

**Published:** 2020-07-17

**Authors:** Ahmed Alfares, Lamia Alsubaie, Taghrid Aloraini, Aljoharah Alaskar, Azza Althagafi, Ahmed Alahmad, Mamoon Rashid, Abdulrahman Alswaid, Ali Alothaim, Wafaa Eyaid, Faroug Ababneh, Mohammed Albalwi, Raniah Alotaibi, Mashael Almutairi, Nouf Altharawi, Alhanouf Alsamer, Marwa Abdelhakim, Senay Kafkas, Katsuhiko Mineta, Nicole Cheung, Abdallah Abdallah, Stine Büchmann-Møller, Yoshinori Fukasawa, Xiang Zhao, Issaac Rajan, Robert Hoehndorf, Fuad Al Mutairi, Takashi Gojobori, Majid Alfadhel

**Affiliations:** 1 Department of Pathology and Laboratory Medicine, King Abdulaziz Medical City, Riyadh, Saudi Arabia; 2 Department of Pediatrics, College of Medicine, Qassim University, Qassim, Saudi Arabia; 3 Qassim University, Department of Pediatrics, Almulyda, Saudi Arabia; 4 Division of Genetics, Department of Pediatrics, King Abdulaziz Medical City, Riyadh, Saudi Arabia; 5 King Abdullah International Medical Research Center, Riyadh, Saudi Arabia; 6 Computer, Electrical & Mathematical Sciences and Engineering Division, Computational Bioscience Research Center, King Abdullah University of Science and Technology (KAUST), Thuwal, 23955-6900 Saudi Arabia; 7 King Saud bin Abdulaziz University for Health Sciences, King Abdulaziz Medical City, Riyadh, Saudi Arabia; 8 Department of Clinical Laboratory Sciences, College of Applied Medical Sciences, King Saud bin Abdulaziz University for Health Sciences, Riyadh, Saudi Arabia; 9 King Abdullah University of Science and Technology (KAUST), Core Labs, Thuwal, 23955-6900 Saudi Arabia; 10 Biological and Environmental Science and Engineering Division, Computational Bioscience Research Center, King Abdullah University of Science and Technology (KAUST), Thuwal, 23955-6900 Saudi Arabia

**Keywords:** Solo, Trio, Trio plus, Whole exome sequencing, Whole genome sequencing, Extending family analysis

## Abstract

**Background:**

Testing strategies is crucial for genetics clinics and testing laboratories. In this study, we tried to compare the hit rate between solo and trio and trio plus testing and between trio and sibship testing. Finally, we studied the impact of extended family analysis, mainly in complex and unsolved cases.

**Methods:**

Three cohorts were used for this analysis: one cohort to assess the hit rate between solo, trio and trio plus testing, another cohort to examine the impact of the testing strategy of sibship genome vs trio-based analysis, and a third cohort to test the impact of an extended family analysis of up to eight family members to lower the number of candidate variants.

**Results:**

The hit rates in solo, trio and trio plus testing were 39, 40, and 41%, respectively. The total number of candidate variants in the sibship testing strategy was 117 variants compared to 59 variants in the trio-based analysis. We noticed that the average number of coding candidate variants in trio-based analysis was 1192 variants and 26,454 noncoding variants, and this number was lowered by 50–75% after adding additional family members, with up to two coding and 66 noncoding homozygous variants only, in families with eight family members.

**Conclusion:**

There was no difference in the hit rate between solo and extended family members. Trio-based analysis was a better approach than sibship testing, even in a consanguineous population. Finally, each additional family member helped to narrow down the number of variants by 50–75%. Our findings could help clinicians, researchers and testing laboratories select the most cost-effective and appropriate sequencing approach for their patients. Furthermore, using extended family analysis is a very useful tool for complex cases with novel genes.

## Background

The advent of next-generation sequencing applications and technologies has provided a low-cost opportunity to examine a patient’s genome and establish molecular defects [1–3]. The clinical evaluation of patients with genetic disorders currently involves whole-exome sequencing (WES) or whole-genome sequencing (WGS), and the diagnostic yield generally ranges between 25 and 49% in some populations, with a maximum yield of 40–44% in trio analysis [4, 5]. The diagnostic yield of WGS ranges between 7 and 20% [6]. Furthermore, family-based analysis and studies of next-generation sequencing provide considerable power to detect common and rare variants [7], and family-based designs can be helpful for cosegregation (e.g., for prioritizing variants and genes) [8]. Additionally, several genes and disorders have been discovered by testing extended consanguineous families [9–11], mainly by autozygome and exome analysis [12]. However, one of the major limitations of these technologies in addition to their cost is the data interpretation. On average, WES costs approximately $1500, and WGS costs approximately $4000; the average number of called variants in the variant call format (VCF) file in WES is approximately 70,000 compared with approximately 5,000,000 in WGS. Therefore, finding the right approach for each case is crucial for clinicians to reach a final diagnosis as well as for the health system to direct its budget by providing the right test. In this study, we examined the best testing strategy by comparing the hit rate of solo cases compared with the rate obtained by extending the test to other family members (trio or trio plus). Furthermore, we analyzed the impact of family structure in cases of trio-based analysis compared with sibship testing. Moreover, we studied the impact of testing extended family members (up to eight members) on narrowing the possible candidate variants, which would require further investigation and manual curation.

## Methods

We retrospectively reviewed all genetic cases that has been seen at the genetic clinic at King Abdulaziz Medical City, Riyadh, Saudi Arabia. Only patients who underwent DNA sequencing; either WES or WGS were recruited for the study. Data collection from patient’s electronic medical records, as well as further analysis were done for eligible patients. Clinical next-generation sequencing based tests (WES and/or WGS) were performed in commercial CAP/CLIA-accredited laboratories. For the extended family analysis, WGS was carried out either in clinical CAP/CLIA-accredited laboratories or in a research laboratory at King Abdullah University of Science and Technology (KAUST) as part of a collaborative project. Illumina NextSeq, Illumina HiSeq or Ion Proton sequencers were used for WES. For WGS, only HiSeq 4000 sequencer were used. Cases who have been tested with WES requires ~90x depth of coverage, and the minimum coverage for any variant to be considered is 20x. The average coverage depth for WGS cases was ~30X. The configuration of the pipeline was based on the sequencing systems and types of kit. Ethical approval for this study was obtained from the Institutional Research Board of the King Abdullah International Medical Research Center (KAIMRC) with a reference study number RC 16/113 and RC16/211/R2.

In order to obtain a diagnosis for cases that has been sent for genetic testing, several factors incorporated in to reach one (or few) pathogenic variants. These factors include but not limited to: 1) the patient clinical presentation, 2) mode of inheritance observed in the family, 3) results of additional metabolic analysis, 3) allele frequency in population databases, and others. When considering testing the extended family to facilitate the analysis; the testing cost and hit rate are the major two considerations.

**Fig. 1:**
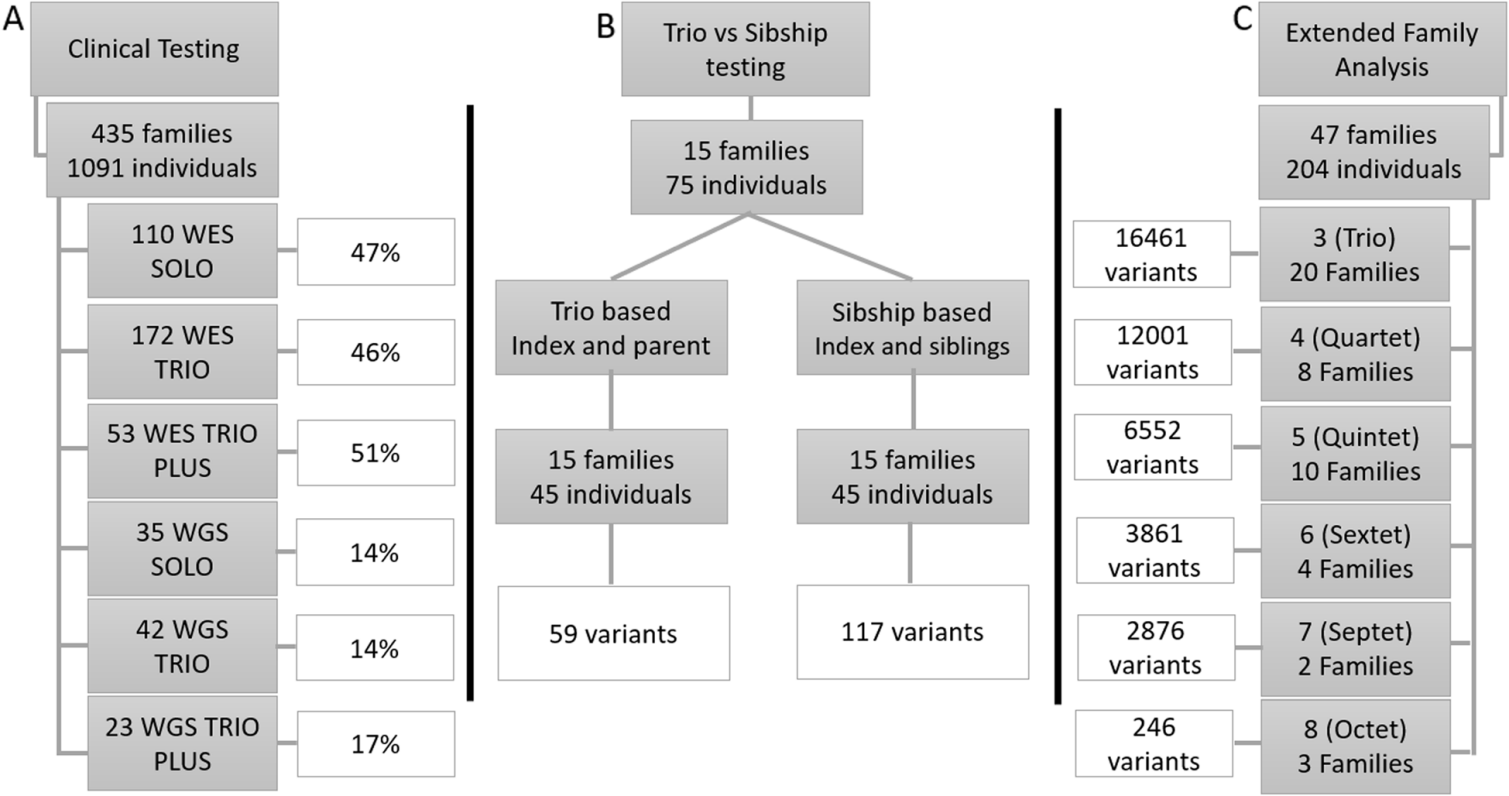
illustration of the three cohort structures enrolled in this study. **a** Clinical testing cohort with the number of enrolled families, individuals and test type; the white rectangle represents the positive results for each test type. **b** Trio vs. sibship cohort, with the number of candidate variants. **c** Extended family analysis cohort; the white rectangle shows the number of candidate variants after adding each family member.

For the different analyses, we divided our main cohort into three cohorts. The first was a clinical cohort used to assess the hit rate of each test type (solo vs. trio vs. trio plus). The second cohort was used to assess the testing strategy between trio-based analysis and siblings only with no parents for the trio vs. sibship-based analysis. The third cohort was employed to assess the impact of testing additional family numbers on the total number of candidate variants for the extended family analysis. Each cohort structure is illustrated in Fig. 1.

### Cohort structures

#### Clinical cohort

In this cohort, families were tested for complete clinical analysis, including variant confirmation and clinical reporting. All clinical cases that underwent WES and/or WGS between 2014 and 2018 were enrolled irrespective of their phenotype. Cases were sorted based on their test type (solo, trio or trio plus), where solo indicates testing performed only in the index case; trio indicates testing performed on the index case and both parents; and trio plus indicates testing performed on the index case, both parents and an additional sibling (either affected or not affected). Finally, we classified each identified variant for clinical significance as pathogenic/likely pathogenic (P/LP), a variant of uncertain significance (VUS) or benign according to the ACMG criteria [13]. Detailed clinical information in the human phenotype ontology (HPO) format and the variant classification of all positive and inconclusive cases are provided in supplementary material Variant Database (Additional file 3). All identified disease-causing variants in this cohort were confirmed by either Sanger sequencing or fragment analysis. Several tools were used for the raw data analysis, including Alamut® Visual (http://www.interactive-biosoftware.com/alamut-visual/), BaseSpace Variant Interpreter (https://variantinterpreter.informatics.illumina.com/), Ingenuity Variant Analysis - QIAGEN Bioinformatics (https://variants.ingenuity.com/va/), Varsome The Human Genomics Community (https://varsome.com/), the UCSC Genome Browser (https://www.genome.ucsc.edu/) and the Integrative Genomics Viewer [3].

#### Trio vs. sibship testing

In this cohort, we enrolled families with a family history suggestive of autosomal recessive disorders and five members (index, both parents and two siblings) for the raw data analysis. Then, we compared the number of coding variants in the same family in the trio-based analysis (index and parents), where the variant was present in both parents in a heterozygous state and in a homozygous state in the index patient, with the sibship-based analysis (index and siblings with no parents), based on health status and the shared and nonshared variants between affected and nonaffected siblings.

#### Extended family analysis cohort

This cohort mixed families from the clinical and other research cohorts to analyze the number of candidate variants based on the family structure using family-based designs (e.g., co-segregation for prioritizing variants and genes). For the extended family analysis, we only used the vcf files generated by the local pipeline. We considered families in which three or more individuals were being tested in this analysis. Candidate variants were considered after annotating the variants as either coding or noncoding (exonic or intronic) variants based on the most severe impact at the mRNA and transcript levels. The total number of identified variants was based on the index case and then narrowed by adding family members (supplementary material Table 2). The extended family analysis focused on the shared and nonshared variants between affected and nonaffected individuals; both homozygous and heterozygous variants were considered.

## Results

### Clinical cohort

The cohort comprised 1091 individuals from 435 families. In 322 families (74%), the index was in the pediatric age group (< 12 years), and in 113 (26%), the index belonged to the adult group; 237 (54%) were male, and 196 (45%) were female. All cases were enrolled through the genetics clinic and received a complete clinical evaluation, including metabolic workup, array comparative genomic hybridization, WES and then WGS if all the previous analyses were negative. In total, 74% were consanguineous families compared with 24% nonconsanguineous, and 2% had an unknown status. The overall hit rate of all the cohorts (WES + WGS) was 40% (WES = 47%, WGS = 15%). The complete breakdown of the results is provided in the supplementary material (Table 1).

**Table 1:** Showing the number of test type (enrolled family members for testing), and the total number of families for each test type for example we have 8 families where we tested 4 individuals (trio plus), 6 individuals underwent WES, and 2 individuals underwent WGS

Test type	Total number of families	WES	WGS
3 (Trio)	20	13	7
4 (Quartet)	8	6	2
5 (Quintet)	10	5	5
6 (Sextet)	4	0	4
7 (Septet)	2	0	2
8 (Octet)	3	0	3

### Hit rate in solo, trio and trio plus

**Fig. 2:**
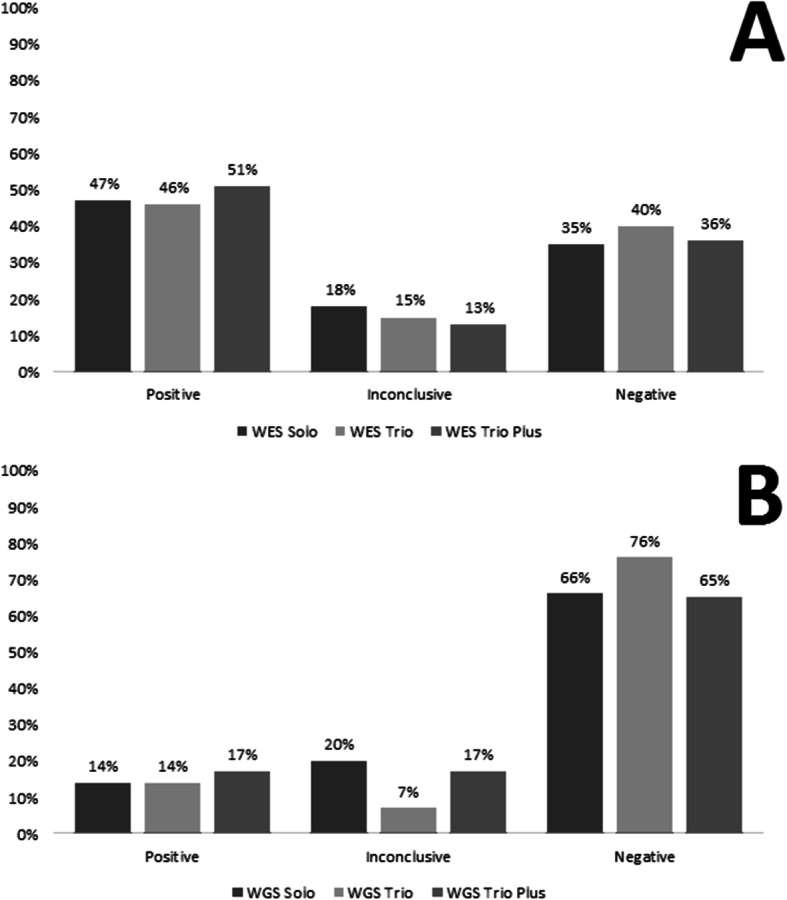
Breakdown of the hit rate by test type. A) for WES and B) for WGS

The total number of WES cases was 335 (110 solo, 172 Trio, 53 Trio plus), of the solo cases, 52 (47%) were positive, compared with 79 (46%) of trio and 27 (51%) of trio plus cases were positive. The total number of WGS cases was 100 (35 solo, 42 Trio, 23 Trio plus), of the solo cases 5 (14%) were positive, compared with 6 (14%) of trio cases, and 4 (17%) of trio plus cases were positive (Fig. 2).

### Trio plus affected and trio plus nonaffected

In total, 76 families underwent trio plus testing, 53 families underwent WES, and 23 families underwent WGS. The WES cases were divided into 41 families with affected individuals and 12 families with unaffected individuals. The hit rate between the two cohorts was 51% (21/41 families) when the trio plus family member was affected and 50% (6/12 families) when the trio plus family member was nonaffected. For the WGS cases (23 families), 15 families had affected individuals, and eight families had unaffected individuals. The hit rate was 13% among the affected plus cohort (2/15 families) and 25% in the nonaffected plus cohort (2/8 families) (supplementary material Table 1).

### Mode of inheritance and testing type

If we consider the mode of inheritance as a clinical guide to testing, among all the positive cases with a family history suggestive of autosomal recessive disorders, the positive results were 70% in solo testing (40 cases), 75% in trio testing (64 cases) and 71% in trio plus testing (22 cases). For families with a suggestive autosomal dominant mode of inheritance, the positive results for solo, trio and trio plus were 25% (14 cases), 20% (17 cases), and 13% (4 cases), respectively (supplementary material file 1:Table 1).

### Sibship testing cohort

For the trio vs. sibship genome, we enrolled families to test the best strategy and compared the trio-based analysis with testing siblings. In particular, we compared trio-based testing where the index and both parents were involved in sibship genome testing without parents. This approach examined the number of candidate variants in the trio-based analysis vs. testing siblings without parents in families with a family history highly suggestive of autosomal recessive disorders to lower the number of candidate variants. For this analysis, we had 15 families with five members, and raw data, detailed family pedigrees (Additional file 2) are provided in the supplementary material. We compared the number of coding variants in the trio-based analysis, where the variant was present in both parents in the heterozygous state and in the index patient in the homozygous state, with testing only siblings without parents. We found that the average number of candidate variants in the trio-based analysis was 59 compared with 117 under the sibship testing strategy (Fig. 1). When we consider if all the siblings are affected (nonaffected), the average number of shared homozygous variants is 90 (88) variants. However, these are the raw results and had limited statistical significance due to the sample size limitation, which occurred because of the testing costs.

### Extended family analysis cohort

**Fig. 3:**
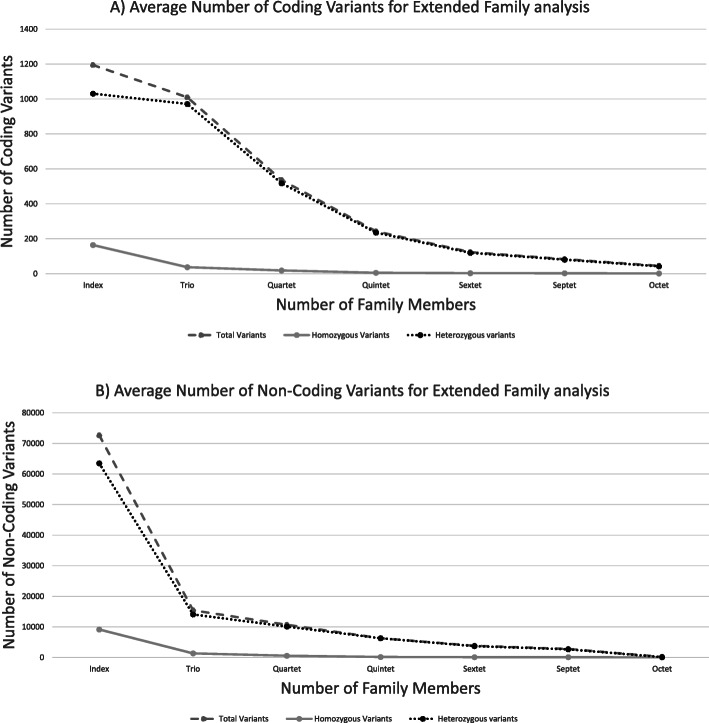
**a** Average number of coding variants for the extended family analysis. **b** Average number of non-coding variants for the extended family analysis

This cohort includes 47 families (204 individuals) (Table 1). For the extended family analysis; we used the VCF files from families to test the impact of testing additional family members to lower the number of candidate variants that would require further analysis. In particular, we assessed shared and nonshared variants among affected and nonaffected family members based on different modes of inheritance and allele state. After applying basic filters, including quality filters and allele frequency < 1.5%, we found 27,646 variants in trio families. We split the analysis into two types: (i) coding and noncoding variants and (ii) homozygous and heterozygous variants. Here, variants on the X chromosome were considered homozygous in cases of males. After we applied filters to look for shared variants between affected members, the number of candidate variants dropped by 50–75% after using the parent results (trio-based analysis) and by 25–50% after adding each additional family member. The average coding homozygous candidate variants dropped to only <= 6 variants after adding the fifth family member and to 2 coding variants after adding the eighth family member. The coding heterozygous variants dropped to 100 variants after adding the sixth family member and to 43 in families with eight family members. The average number of noncoding variants per index was 72,660 after passing the threshold for quality and allele frequency. However, after adding the eighth family member, the number of noncoding variants dropped to 66 homozygous and 135 heterozygous variants (Table 2, Fig. 3).

**Table 2:** Showing the average number of variants either shared or not shared after adding each family member (complete details about filtration process of shared not shared variants are provided in supplementary material file 1:Table 2)

Coding			
Number of tested individuals	Total Variants	Homozygous Variants	Heterozygous Variants
Index	1196	165	1031
Trio	1010	38	972
Quartet	591	20	571
Quintet	242	6	236
Sextet	104	4	100
Septet	84	3	81
Octet	45	2	43
Non-Coding			
Number of tested individuals	Total Variants	Homozygous Variants	Heterozygous Variants
Index	72,660	9162	63,498
Trio	15,451	1361	14,090
Quartet	11,410	660	10,750
Quintet	6309	174	6135
Sextet	3757	113	3644
Septet	2792	111	2681
Octet	201	66	135

## Discussion

In this study, we examined the advantages of additional family members. Previously, we noticed no differences in the hit rate between solo and trio in our population, and we wanted to further test this observation [3, 4]. In this analysis, we confirmed that the hit rate between solo and other extended family analyses has limited clinical utility. One possible explanation is that the majority of the disease-causing variants in our population were homozygous in the autosomal recessive disorder where both parents were carriers. Furthermore, autosomal dominant disorders account for only 10% of all detected disorders in genetics clinics [4, 5], and our cohort of both adult and pediatric cases would capture autosomal dominant late-onset disorders. However, even with similar hit rates, one of the advantages of trio or trio plus testing over solo testing is reducing the turnaround time and providing results faster than by performing segregation analysis for the candidate variants.

After considering family history as a clinical tool to determine the testing type, we found no differences in the hit rate (solo vs. trio vs. trio plus) between families with autosomal recessive disorders and families with autosomal dominant disorders. This extended testing showed no additional advantages for the hit rate, but it might provide useful information for databasing.

In large families, it is always challenging for clinical geneticists and genetics counselors to decide who to test. To further evaluate the impact of additional family members, we tested the power of lowering the possible candidate variants that would require further evaluation. For homozygous variants that support autosomal recessive disorders, testing parents under the traditional trio-based approach would provide a lower number of candidate variants compared with sibship testing with no parents.

To test the impact of adding family members to the number of candidate variants, we found that each additional family member could lower the number of candidate variants in the coding region by 25–50%. Hence, while this method might be expensive, we could solve two cases and establish the molecular defect (underwork in different research project). However, this approach might not always explain the phenotype if the variants are in genes unrelated to the phenotype or if the variants are in novel genes, and further research is required to investigate the gene function and relation with the phenotype.

In addition, we previously showed the limited clinical utility of WGS compared with WES [5]. In this study, we confirmed our previous observations. Indeed, of all 14 of the cases identified by WGS, the variant was called in the vcf files from the exome in 13 cases. One possible explanation for overlooking these cases during the analysis rather than the advantages of WGS is that we tend to consider WGS as a more rigorous form of testing. Moreover, we examine the VCF file in more detail than with WES data, which normally passes through the routine pipeline of clinical evaluation. We re-emphasize our previous recommendation to reanalyze WES before performing WGS. This would improve the hit rate of WES and save a significant amount of money. For example, in one case, we performed WGS trio plus, which cost approximately $13,000, and we identified a missense variant that was found by WES but overlooked during the analysis; in another example, we spent approximately $100,000 on only three families to perform WGS for all eight family members. This amount could be saved, and we could test sixty-six families for solo WES and reach a similar diagnostic hit rate. Furthermore, the average number of total called variants in WGS is 5,000,000 per individual, and even with trio or trio plus family analysis, the average number of identified variants would drop to 8000–15,000 after applying all routine filters (allele frequency, low quality, shared and nonshared variants), with approximately 96–97% being intronic variants. With the current stage of knowledge, it is not possible to examine this large number of variants, and testing laboratories focus only on 2–3%, which covers the coding exonic part of the genome, or look for previously reported intronic variants. Therefore, until the price of genomes falls or knowledge and databases grow to increase our understanding of the noncoding regions for analysis and classification, we recommend performing WES and a reanalysis of the raw data from WES.

While solo exome testing might provide the best clinical practice in our population, there might be other scenarios in which extended analysis is required, such as when results are urgently required for medical or nonmedical reasons, when other family members might not be available in future visits or when segregation analysis for any identified variant is not possible.

## Conclusion

There was no difference in the hit rate between solo and extended family members. Trio-based analysis was a better approach than sibship testing. However, each additional family member helped to narrow down the number of variants by 50–75%. These findings could help clinicians, researchers and testing laboratories select the most cost-effective and appropriate sequencing approach for their patients. As with any research, some potential limitations should be further studied in future researches. For instance, this study needs to be extend to wider range of individuals as our study is limited by a small sample size due to the nature and cost of WES and WGS.

## Supplementary information

**Additional file 1.**

**Additional file 2.**

**Additional file 3.**

## Data Availability

The authors declare that the data supporting the findings of this study is available within the paper and its supplementary information files. All variants identified through this study is available in ClinVar database and accession numbers are provided for each variant in the supplementary documents (Additional file 3). All variants has been submitted by the study group under this link https://www.ncbi.nlm.nih.gov/clinvar/submitters/506517/. The raw datasets generated during the current study are not publicly available because it is possible that individual privacy could be compromised. This risk was noted by the ethics committee that approved our study and one of the conditions of approval was that the raw data could not be made publicly available.

## Abbreviations

WES: Whole-Exome Sequencing
WGS: Whole-Genome Sequencing
VCF: Variant Call Format
CAP: College of American Pathologists
CLIA: Clinical Laboratory Improvement Amendments
KAUST: King Abdullah University of Science and Technology
P: Pathogenic
LP: Likely Pathogenic
VUS: Variant of Uncertain Significance
ACMG: American College of Medical Genetics and Genomics
HPO: Human Phenotype Ontology
