# Supplementary material for: What is the right sequencing approach? Solo VS extended family analysis in consanguineous populations
Source: BMC Med Genomics. 2020 Jul 17;13:103. doi: 10.1186/s12920-020-00743-8 (PMC7368798; doi:10.1186/s12920-020-00743-8)
Supplement: Supplementary file 1 — Additional file 1. [file 12920_2020_743_MOESM1_ESM.docx]

Table 1: Breakdown of results of the clinical testing cohort.

| Total Number of Individuals in the Clinical Cohort  Total Number of Indexes | | | | | | | 1091  435 |
| --- | --- | --- | --- | --- | --- | --- | --- |
|  |  | **Adults** | 113 | **Peds** | 322 |  |  |
|  | **Males** | 237 | **Females** | 196 | **Fetus** | 2 |  |
|  | Consanguinity | | | | | |  |
| Yes | | | 322 | | 74% | | |
| No | | | 105 | | 24% | | |
| NA | | | 8 | | 2% | | |
|  | Hit Rates | | | | | |  |
|  | Positive | | Inconclusive | | Negative | | Total |
| Overall | 173 | | 66 | | 196 | | 435 |
|  | 40% | | 15% | | 45% | | 100% |
| WES vs. WGS | | | | | | | |
| WES | 158 | | 52 | | 125 | | 335 |
|  | 47% | | 16% | | 37% | | 100% |
| WGS | 15 | | 14 | | 71 | | 100 |
|  | 15% | | 14% | | 71% | | 100% |
| by Consanguinity | | | | | | | |
| Yes | 128 | | 50 | | 144 | | 322 |
|  | 40% | | 16% | | 45% | | 100% |
| No | 39 | | 15 | | 51 | | 105 |
|  | 37% | | 14% | | 49% | | 100% |
| NA | 6 | | 1 | | 1 | | 8 |
|  | 75% | | 13% | | 13% | | 100% |
| Solo | | | | | | | |
| WES | 52 | | 20 | | 38 | | 110 |
|  | 47% | | 18% | | 35% | | 100% |
| WGS | 5 | | 7 | | 23 | | 35 |
|  | 14% | | 20% | | 66% | | 100% |
| Total | 57 | | 27 | | 61 | | 145 |
|  | 39% | | 19% | | 42% | | 100% |
| Trio | | | | | | | |
| WES | 79 | | 25 | | 68 | | 172 |
|  | 46% | | 15% | | 40% | | 100% |
| WGS | 6 | | 3 | | 33 | | 42 |
|  | 14% | | 7% | | 79% | | 100% |
| Total | 85 | | 28 | | 101 | | 214 |
|  | 40% | | 13% | | 47% | | 100% |
| Trio Plus | | | | | | | |
| WES | 27 | | 7 | | 19 | | 53 |
|  | 51% | | 13% | | 36% | | 100% |
| WGS | 4 | | 4 | | 15 | | 23 |
|  | 17% | | 17% | | 65% | | 100% |
| Total | 31 | | 11 | | 34 | | 76 |
|  | 41% | | 14% | | 45% | | 100% |
| Trio Plus Affected | | | | | | | |
| WES | 21 | | 6 | | 14 | | 41 |
|  | 51% | | 15% | | 34% | | 100% |
| WGS | 2 | | 4 | | 9 | | 15 |
|  | 13% | | 27% | | 60% | | 100% |
| Total | 23 | | 10 | | 23 | | 56 |
|  | 41% | | 18% | | 41% | | 100% |
| Trio Plus Unaffected | | | | | | | |
| WES | 6 | | 1 | | 5 | | 12 |
|  | 50% | | 8% | | 42% | | 100% |
| WGS | 2 | | 0 | | 6 | | 8 |
|  | 25% | | 0% | | 75% | | 100% |
| Total | 8 | | 1 | | 11 | | 20 |
|  | 40% | | 5% | | 55% | | 100% |
| Inheritance | | | | | | | |
| AR | 126 | | 36 | | - | | 162 |
| AD | 35 | | 26 | | - | | 61 |
| XLD | 0 | | 0 | | - | | 0 |
| XL | 12 | | 3 | | - | | 15 |
| NA | 0 | | 1 | | - | | 1 |
| Total | 173 | | 66 | | - | | 239 |
| AR | **AD** | | **XLD** | | **XL** | | **Total** |
| Solo | | | | | | | |
| 40 | 14 | | 0 | | 3 | | 57 |
| 70% | 25% | | 0% | | 5% | | 100% |
| Trio | | | | | | | |
| 64 | 17 | | 0 | | 4 | | 85 |
| 75% | 20% | | 0% | | 5% | | 100% |
| Trio Plus | | | | | | | |
| 22 | 4 | | 0 | | 5 | | 31 |
| 71% | 13% | | 0% | | 16% | | 100% |
|  | **Solo** | | **Trio** | | **Trio Plus** | | **Total** |
| Autosomal Dominant | | | | | | | |
|  | 40 | | 64 | | 22 | | 126 |
|  | 32% | | 51% | | 17% | | 100% |
| Autosomal Recessive | | | | | | | |
|  | 14 | | 17 | | 4 | | 35 |
|  | 40% | | 49% | | 11% | | 100% |

Table 2: Filtration strategy for extended family analysis to narrow the number of candidate variants

| **Total number of variants** |
| --- |
| a. Total coding and non-coding variants in index only  b. Total shared coding and non-coding variants in trio (present in index and either father or mother or both)  c. Total shared variants after adding each affected additional family member (present in above and the additional member)  d. (Repeat c for each additional family member) |
| **For shared homozygous variants** |
| a. Total coding and non-coding homozygous variants in index  b. a + heterozygous variants in both parents  c. For each additional family member if affected, count shared homozygous  OR d. For each additional family member if not-affected, remove shared coding and non-coding homozygous variants. |
| **For shared heterozygous variants** |
| a. Total coding and non-coding heterozygous variants in index  b. Total shared coding and non-coding heterozygous variants in index and heterozygous in one or both parents  c. For each additional family member if affected, count shared coding and non-coding heterozygous variants  OR d. For each additional family member if not-affected, remove shared coding and non-coding heterozygous variants. |
