# Supplementary material for: What is the right sequencing approach? Solo VS extended family analysis in consanguineous populations
Source: BMC Med Genomics. 2020 Jul 17;13:103. doi: 10.1186/s12920-020-00743-8 (PMC7368798; doi:10.1186/s12920-020-00743-8)
Supplement: Supplementary file 2 — Additional file 2. [file 12920_2020_743_MOESM2_ESM.pdf]

Family 1:

Multiple anomalies in the spine (HP:0000925), Abnormality of the thoracic spine (HP:0100711).

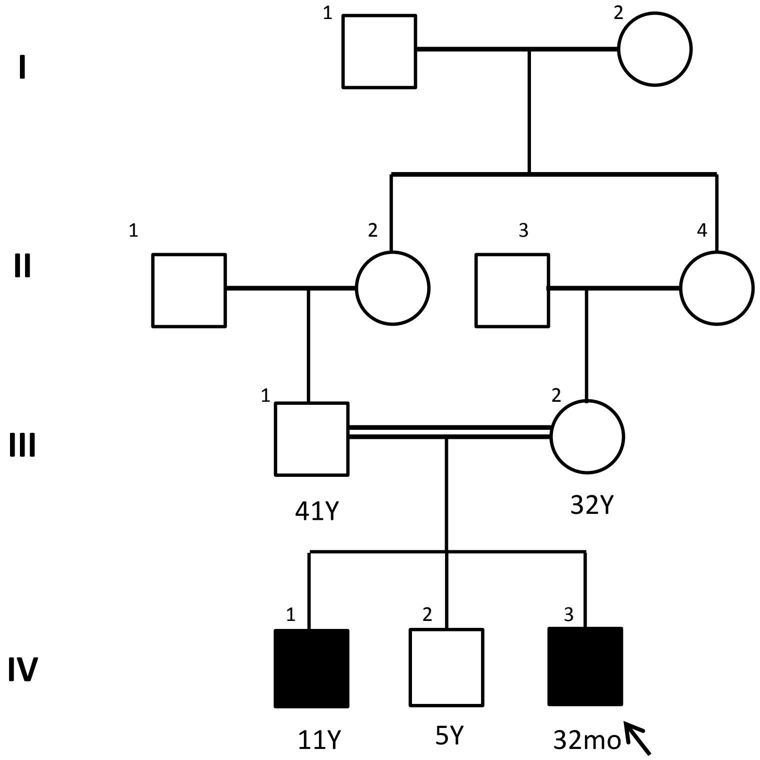

Family 2:

■ Celiac disease(HP:0002608), Diabetes mellitus(HP:0000819), Triangular face(HP:0000325), prominent forehead(HP:0011220), Jaundice (HP:0000952), Failure to thrive(HP:0001508), Cholestasis (HP:0001396)

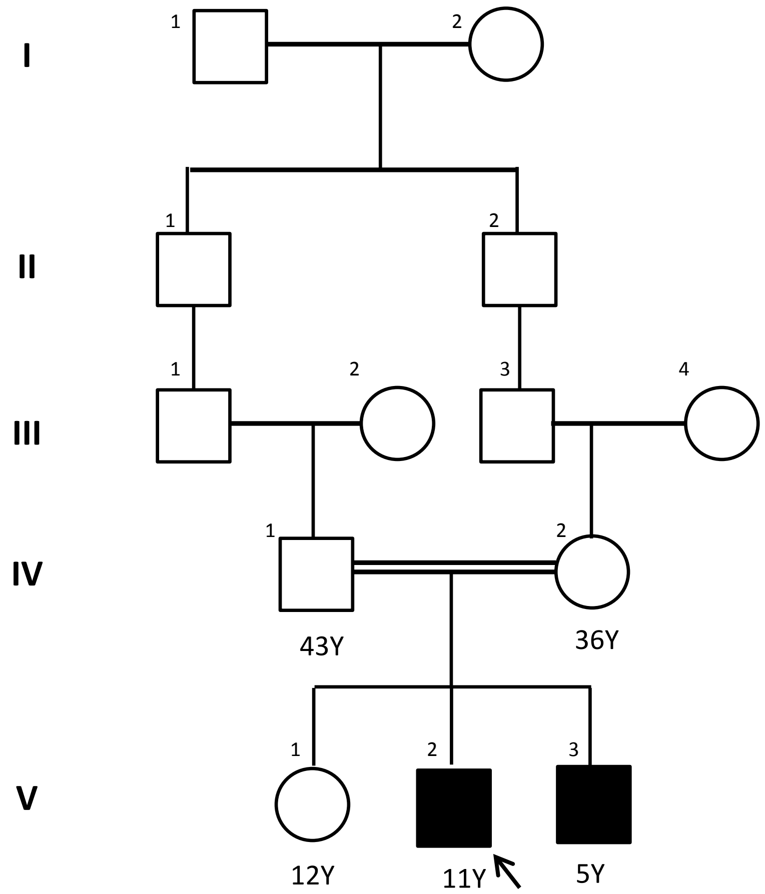

Family 3:

Speech delay (HP:0000750) hyperactivity (HP:0000752), Intellectual Disability (HP:0001249), frontal bossing (HP:0002007), low nasal root (HP:0005280), clinodactyly of fourth finger (HP:0040025)

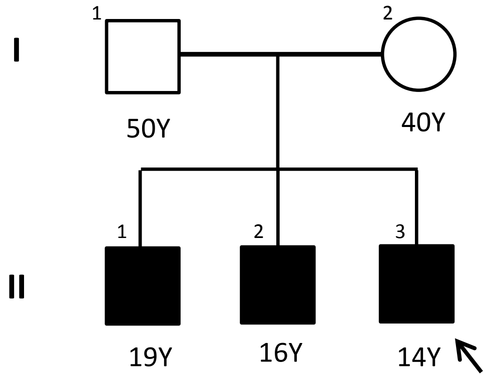

Family 4:

Congenital hypothyroidism (HP:0000851), hypermetropia (HP:0000540), decreased fetal movement (HP:0001558), wide distal phalenx (HP:0009642) ,dry skin (HP:0000958), Nystagmus (HP:0006934),

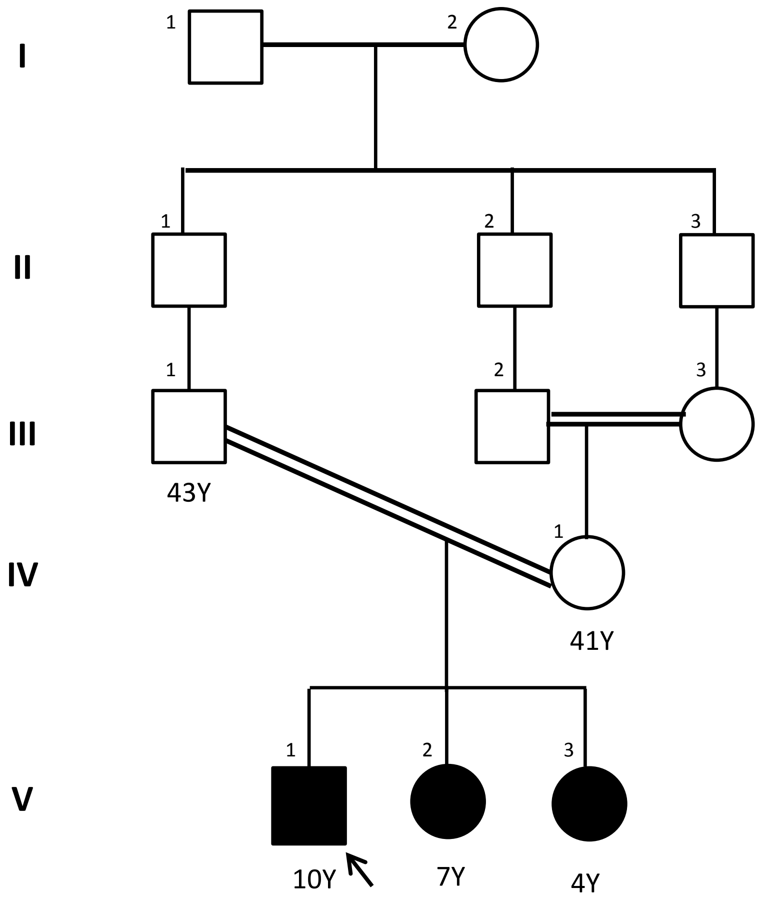

Family 5:

Global Developmental delay (HP:0001263), worsening scoliosis (HP:0008458), atrial septal defect (HP:0001631), spastic diplegia (HP:0001264).

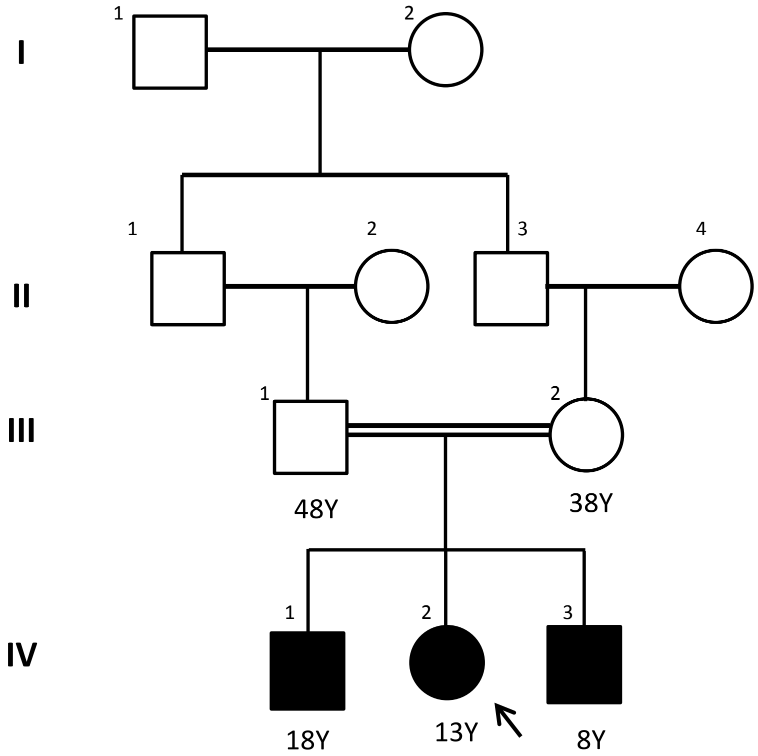

Family 6:

Hypotonia. (HP:0001290), Developmental delay (HP:0001263), Infantile spasms (HP:0012469), strabismus (HP:0000486), and seizures (HP:0001250).

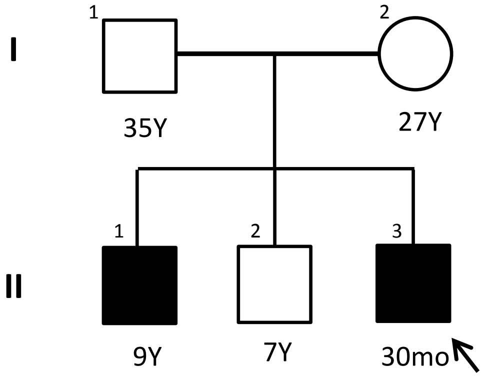

Family 7:

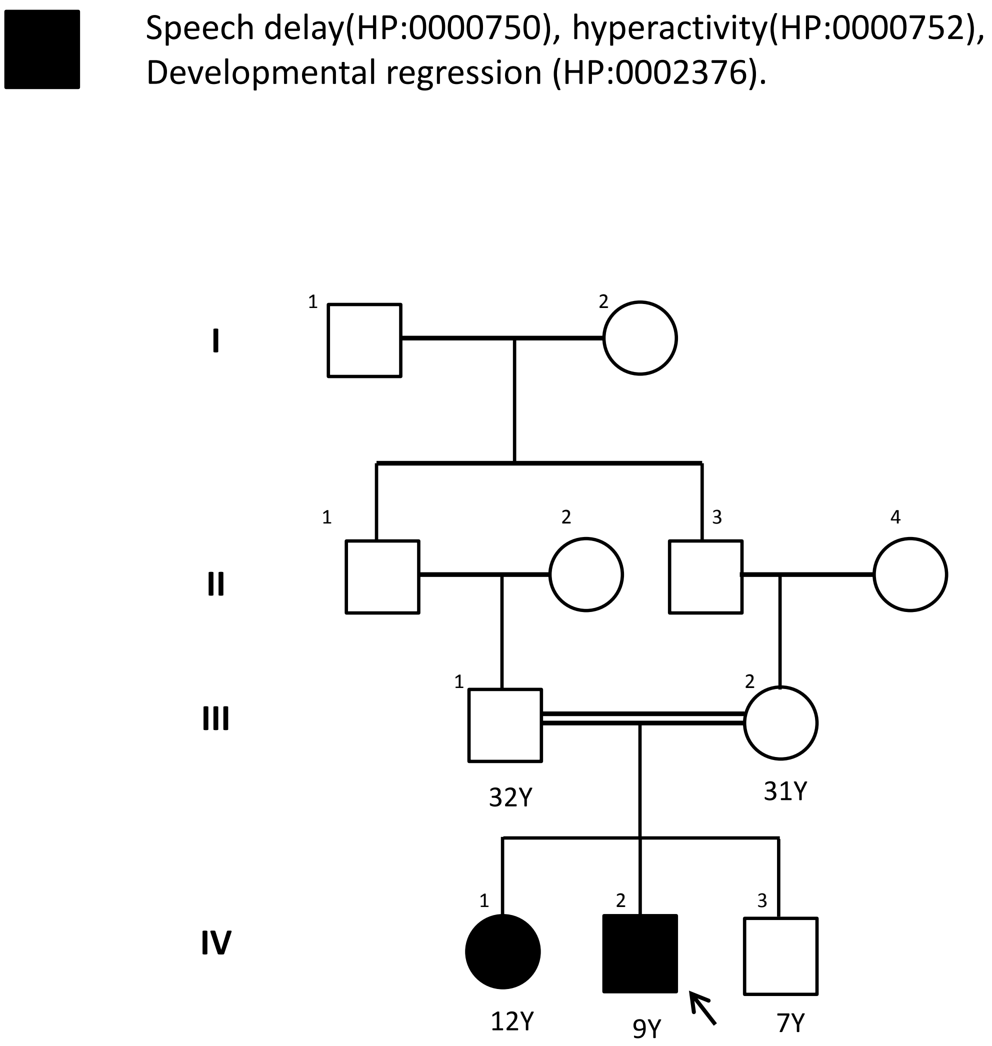
Speech delay(HP:0000750), hyperactivity(HP:0000752),
Developmental regression (HP:0002376).
I
II
III
IV
1
2
1
2
3
4
1
2
32Y
31Y
1
2
3
12Y
9Y
7Y

Family 8:

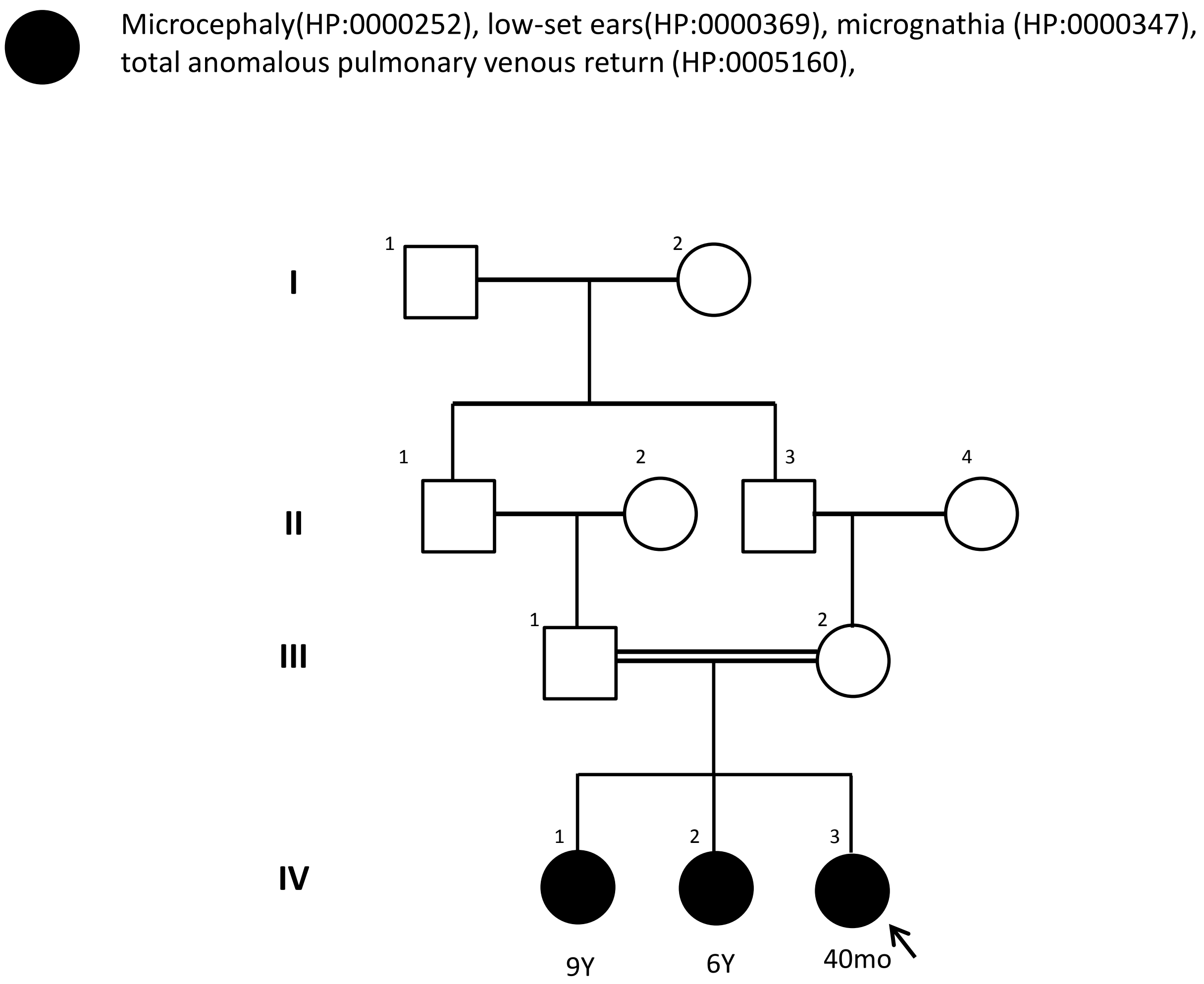

Microcephaly(HP:0000252), low-set ears(HP:0000369), micrognathia (HP:0000347),
total anomalous pulmonary venous return (HP:0005160),
I
II
III
IV
1
2
1
2
3
4
1
2
1
2
3
9Y
6Y
40mo

Family 9:

Speech delay (HP:0009889), anxiety (HP:0000739), hyperactivity (HP:0000752), febrile convulsion (HP:0002373), fetal distress (HP:0025116), large for gestational age (HP:0001520),Developmental delay (HP:0001263), Specific learning disability (HP:0001328).

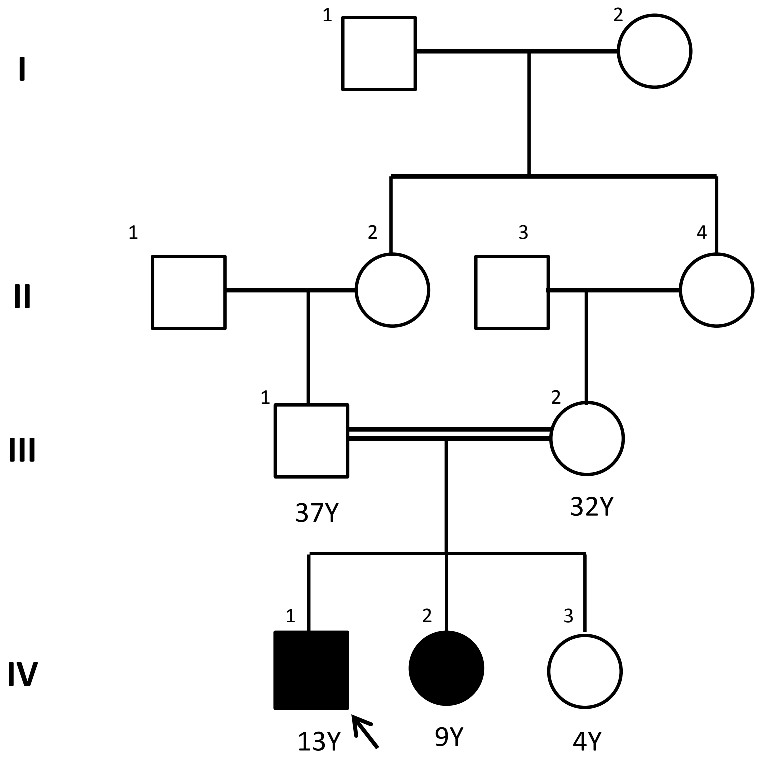

Family 10:

Multiple anomalies in the spine (HP:0000925), abnormality of the thoracic spine (HP:0100711).

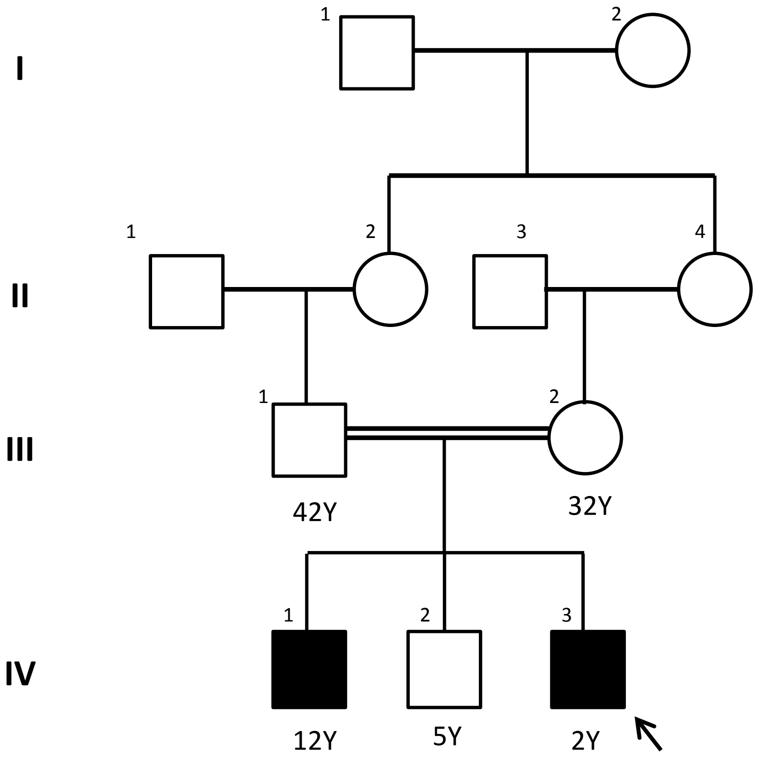

Family 11:

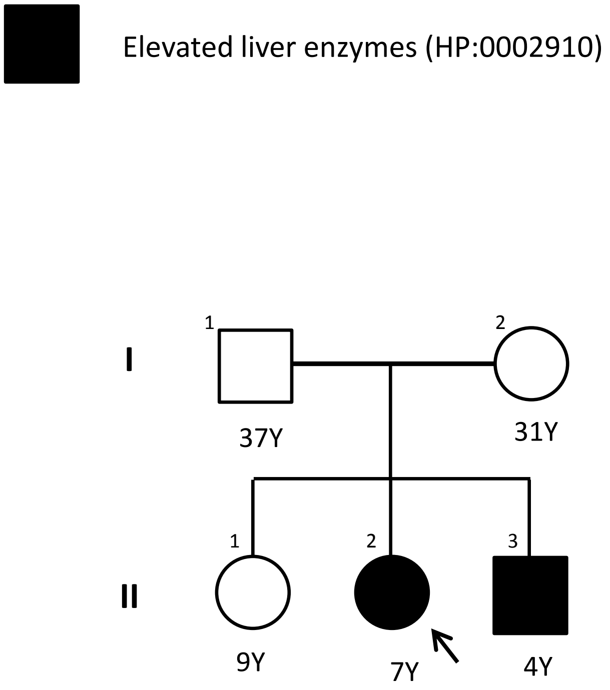

Family 12:

Strokelike episodes (HP:0002401), headache (HP:0002315), vomiting (HP:0002013), abdominal pain (HP:0002027), progressive encephalopathy (HP:0002448), Spastic quadriplegia (HP:0002510),

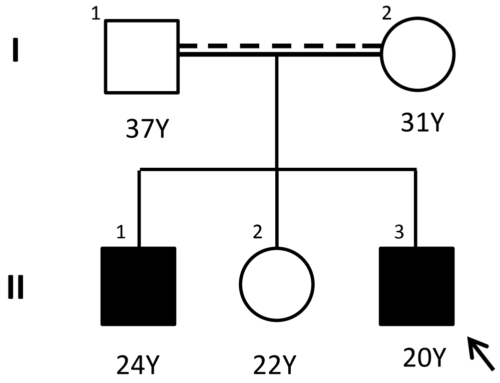

Family 13:

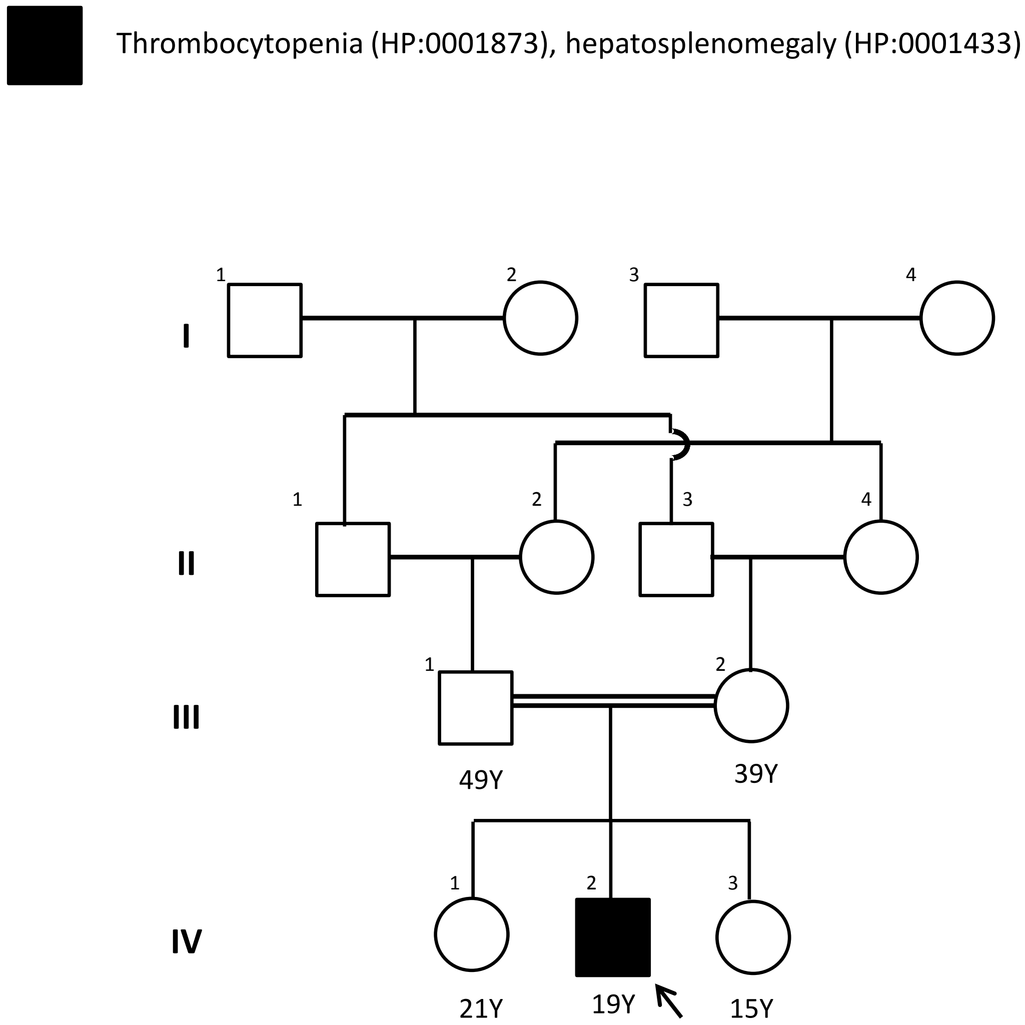

Thrombocytopenia (HP:0001873), hepatosplenomegaly (HP:0001433)
I
II
III
IV
1
2
3
4
1
2
3
4
1
2
49Y
39Y
1
2
3
21Y
19Y
15Y

Family 14:

■ ataxia (HP:0001251), global developmental delay (HP:0001263), bronchial asthma (HP:0002099), squint (HP:0000486), protruding ears (HP:0000411), widely spaced teeth (HP:0000687) ,uplifted earlobe (HP:0009909), long philtrum (HP:0000343)

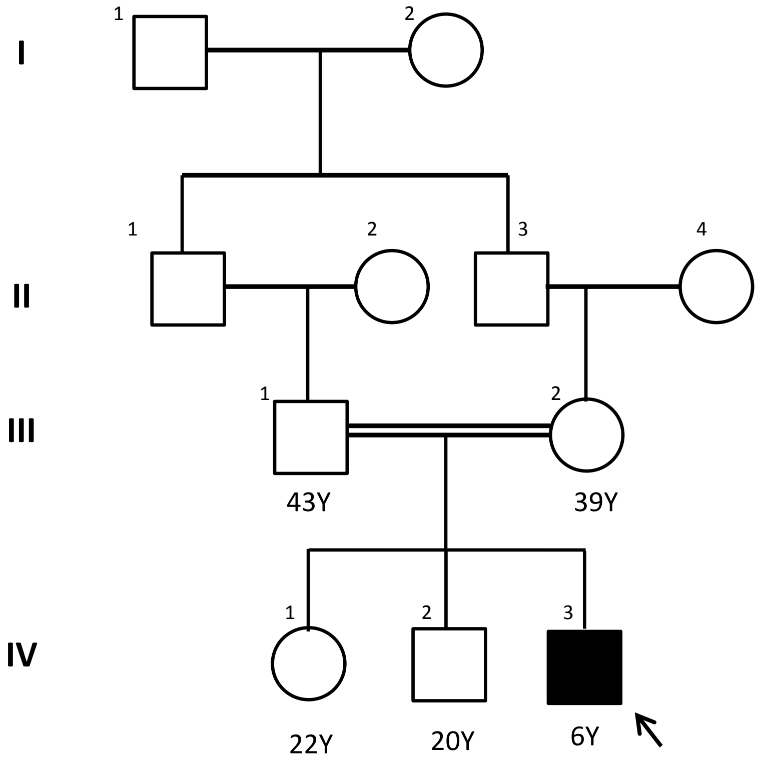

Family 15:

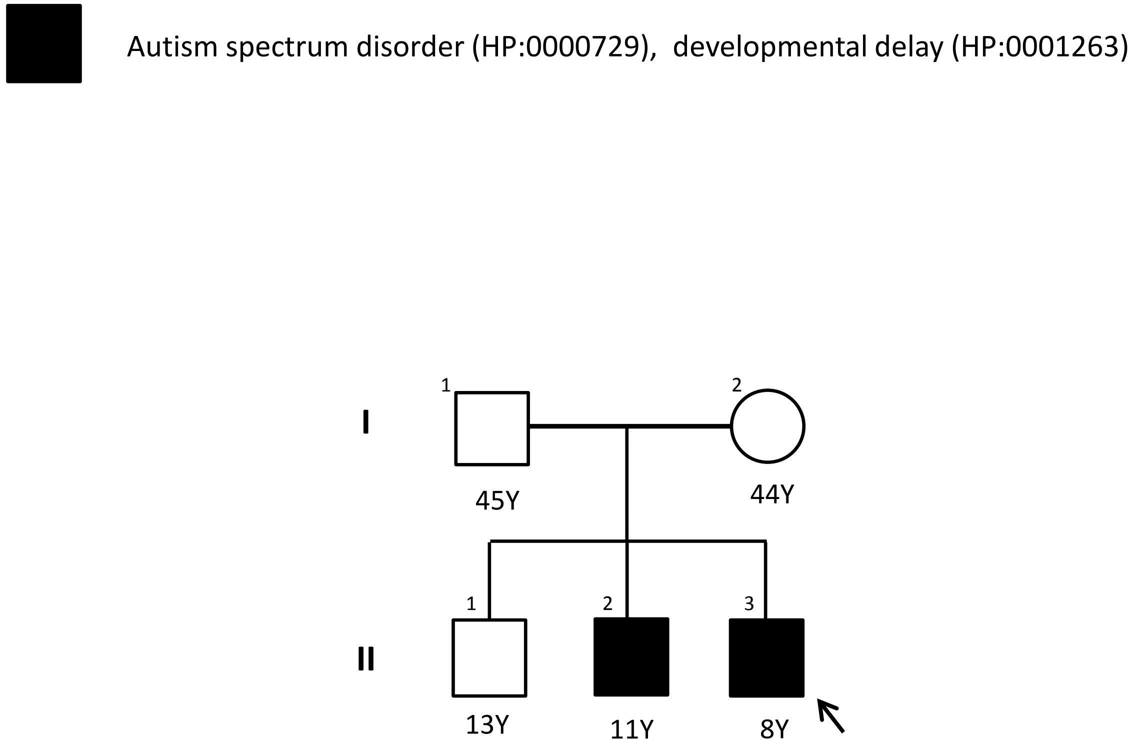
Autism spectrum disorder (HP:0000729), developmental delay (HP:0001263)
I
II
1
2
45Y
44Y
1
2
3
13Y
11Y
8Y
